# H4K20me2: Orchestrating the recruitment of DNA repair factors in nucleotide excision repair

**DOI:** 10.1080/19491034.2018.1444327

**Published:** 2018-03-27

**Authors:** Shalaka Chitale, Holger Richly

**Affiliations:** a Laboratory of Molecular Epigenetics, Institute of Molecular Biology (IMB), Mainz, Germany; b Faculty of Biology, Johannes Gutenberg University, Mainz, Germany

**Keywords:** DNA repair, nucleotide excision repair, MMSET, DICER, H4K20me2

## Abstract

The integrity of the genome is maintained by specific DNA repair pathways. The main pathway removing DNA lesions induced by exposure to UV light is nucleotide excision repair (NER). The DNA damage response at chromatin is accompanied by the recruitment of DNA repair factors to the lesion site and the deposition of specific histone marks. The function of these histone marks in NER stays for the most part elusive. We have recently reported that the methyltransferase MMSET catalyzes the dimethylation of histone H4 at lysine 20 (H4K20me2) at the lesion site. The deposition of H4K20me2 at DNA damage sites elicits the recruitment of the NER factor XPA providing evidence for an H4K20me2-dependent DNA repair factor recruitment mechanism during lesion recognition in the global-genomic branch of NER. Here we discuss how H4K20me2 might impact on the chromatin conformation and the DNA damage response.

One of the unanswered questions in nucleotide excision repair concerns the specific recruitment of DNA repair factors to chromatin. Many reports have addressed the sequential recruitment of factors, but not much is known about the role of histone modifications in this process. The Repair-Prime-Access model [1] suggests that DNA repair consists of three major steps, decondensation of chromatin, access of the repair machinery and finally repair. Nucleotide excision repair may result in chromatin decondensation of several kilobases of DNA around the lesions site [2]. In addition, studies have shown that there is a global relaxation of chromatin in nuclei exposed to localized UV-C damage [3]. The end of the repair process is characterized by restoration of the nucleosome structure of the repaired DNA through interactions of late operating NER proteins and histone chaperones, and results in deposition of histone H3.1 at the site of repair [4]. Additionally, nucleosome replacement must be accompanied by a mechanism of resetting of the histone modifications within chromatin [5]. These rearrangements of chromatin are facilitated by specific chromatin remodelers and presumably also histone modifications. Several studies point at a role for ATP dependent remodelers in the initiation of NER [6] and in the unwinding of DNA. One striking example is Transcription Factor IIH (TFIIH), which opens the DNA helix whereupon RPA and XPA bind and stabilize the repair complex. XPA is one of the fundamental factors operating in NER and is essential for faithful repair [7]. Patients lacking functional XPA show almost no repair of UV lesions and have a very high risk of developing skin cancer [8]. XPA forms a critical link between the recognition of the lesion, modifications in chromatin structure [9], and recruitment of XPB, XPF, XPG and the entire core repair machinery that can excise and resynthesize the damaged stretch of DNA. The factors involved in recruitment and stabilization of XPA, however, remain largely unclear. Several proteins have been shown to play a role in XPA recruitment, with the most important being the TFIIH protein complex [10]. XPA interacts with both the damage recognition machinery i.e. proteins like DDB2 and XPC, as well as the downstream repair machinery i.e factors like RPA and XPF. Additionally, XPA also interacts with certain components of the chromatin remodeling machinery. One of the best characterized interactions of XPA with chromatin modifiers, is its interaction with PARP1 [11]. PARP1 activation is required for decondensation of chromatin in response to UV damage. However, beyond this interaction, the interaction of XPA with chromatin and histone modifications has not been extensively studied. Considering the critical function of XPA in the NER recruitment cascade, studying additional mechanisms of XPA binding and recruitment to DNA should be of great interest.

We have recently reported that a specific chromatin mark, H4K20me2, promotes the recruitment of XPA to the DNA damage site (Figure 1). Our previous work had demonstrated that the endoribonuclease DICER plays an important role in chromatin decondensation during NER [12]. Interestingly, this novel function of DICER is completely independent of its enzymatic activity. DICER associates with PARP1 and the DICER-mediated chromatin remodelling function is strictly dependent on PARP1 activation [12]. To better understand how DICER impacts on chromatin, we studied whether histone modifications are affected by DICER dependent chromatin decondensation. DICER recruits MMSET, a histone methyltransferase, to the DNA damage site [13]. MMSET is capable of setting the H3K36me2 and H4K20me2 marks, depending on the chromatin context. In response to UV damage and in association with DICER, MMSET sets the H4K20me2 mark, which is required for efficient recruitment of XPA to sites of DNA lesions [13]. However, XPA does not inherently contain any methyl-binding domains. Interestingly, the domain of XPA that is essential for its recruitment to H4K20me2 is responsible for the interaction of XPA with RPA2/32. Additionally, 53BP1, a known interactor of H4K20me2, is also required to mediate the interaction of XPA with H4K20me2. Depletion of 53BP1, via siRNA knockdown, resulted in an impaired recruitment of XPA to the H4K20me2 mark. We also confirmed that XPA and 53BP1 interact when stabilized by RPA2/32. Thus, our work elucidates a novel mechanism linking XPA recruitment to chromatin and histone modifications.

**Figure 1.**
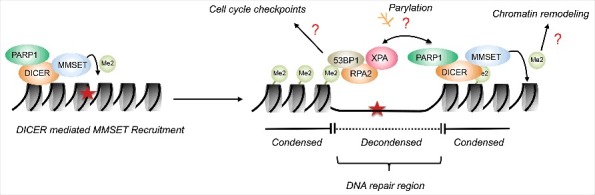
Potential roles of DICER and MMSET mediated H4K20me2 in nucleotide excision repair. UV damage results in recruitment of DICER to the DNA lesion. DICER mediates chromatin decondensation via PARP1 activation, and setting of the H4K20me2 mark via MMSET recruitment. This may further lead to cell cycle regulation via 53BP1 binding, limiting of DNA decondensation via methylation of H4K20, and potentially reciprocal regulation of XPA and PARP1 binding via changes in parylation.

This study raises several interesting avenues for future investigation. XPA has been shown to play a role in positioning and formation of the repair bubble [14]. The decondensation role of DICER may be required for formation of the repair bubble. Additionally, H4K20me2, and recruitment of its interactors, might be essential for positioning of the bubble. 53BP1 seems to bridge the interaction between H4K20me2 and XPA in NER. 53BP1 is an important protein in DSB repair, and promotes non-homologous end joining (NHEJ) over homologous recombination (HR) [15]. It is one of the first proteins recruited to the damage site, and its recruitment is dependent on H2AX phosphorylation, H2A ubiquitylation at lysine 15 and H4K20me2. 53BP1 is part of the universal DNA damage response and is involved in DNA damage checkpoint control. It regulates the phosphorylation of ATM substrates such as CHK2 and SMC1 [16] and is required for accumulation of p53 in response to IR [17,18]. The interaction of XPA and 53BP1 at H4K20me2 decorated chromatin could possibly serve as a sensor for residual DNA damage or as a signal for stalled repair. During DSB repair, 53BP1 is recruited to DNA break sites, and this leads to the accumulation of p53 promoting further downstream cell cycle signaling. We hypothesize that recruitment of 53BP1 to sites of UV damage via H4K20me2 binding could lead to a similar p53 accumulation. In this case, 53BP1 may function as a sensor of multiple types of DNA damage. Nucleosomal displacement during the further steps of repair may ensure loss of 53BP1 and signal completion of the repair process. Further, the process of MMSET recruitment and H4K20me2 methylation could restrict the chromatin decondensation initiated by DICER. Our unpublished data shows that in absence of MMSET, DICER dependent decondensation increases significantly. UV damage can lead to decondensation of several kilobases of DNA at the lesion site and potentially cause a global relaxation of DNA. The mechanisms regulating the extent of decondensation are not well understood. An interesting question is whether the spread of chromatin disruption is regulated by organization of chromatin into functional domains. It was recently shown that CTCF, a protein that regulates boundaries between heterochromatin and euchromatin, is recruited to sites of DNA damage through parylation [19]. Hence, MMSET dependent setting of H4K20 dimethylation could possibly provide an additional mechanism of compartmentalization or regulation of chromatin decondensation. Furthermore, XPA is a critical factor for the progression of efficient NER. Thus, it might seem a good strategy to have multiple redundant pathways of XPA recruitment. Presence of the recognition factor XPC is required for recruitment of XPA to chromatin. Similarly, XPC is also required for ZRF1 recruitment [20,21], and thus presumably for DICER recruitment and setting of H4K20me2. On the other hand, TFIIH is not required for DICER recruitment, and it is also not recruited to the H4K20me2 marked chromatin [13]. Thus, XPC, H4K20me2 and TFIIH might provide a reinforcement of XPA recruitment during different steps of the repair machinery assembly. It is also possible that XPC-, TFIIH- and H4K20me2- dependent XPA recruitment operate in parallel. Thus, absence of one of these recruitment mechanisms would not result in a complete loss of XPA at the damage site.

In conclusion, the elucidation of chromatin based XPA recruitment mechanisms might provide important new insights into the regulation of the chromatin landscape during DNA damage repair. It could also serve as a potential link between chromatin structure and the universal DNA damage response.
